# A fused-image-based approach to detect obstructive sleep apnea using a single-lead ECG and a 2D convolutional neural network

**DOI:** 10.1371/journal.pone.0250618

**Published:** 2021-04-26

**Authors:** S. M. Isuru Niroshana, Xin Zhu, Keijiro Nakamura, Wenxi Chen

**Affiliations:** 1 Biomedical Information Engineering Lab, The University of Aizu, Aizuwakamatsu, Fukushima, Japan; 2 Division of Cardiovascular Medicine, Ohashi Medical Center, Toho University, Meguro, Tokyo, Japan; Taipei Medical University, TAIWAN

## Abstract

Obstructive sleep apnea (OSA) is a common chronic sleep disorder that disrupts breathing during sleep and is associated with many other medical conditions, including hypertension, coronary heart disease, and depression. Clinically, the standard for diagnosing OSA involves nocturnal polysomnography (PSG). However, this requires expert human intervention and considerable time, which limits the availability of OSA diagnosis in public health sectors. Therefore, electrocardiogram (ECG)-based methods for OSA detection have been proposed to automate the polysomnography procedure and reduce its discomfort. So far, most of the proposed approaches rely on feature engineering, which calls for advanced expert knowledge and experience. This paper proposes a novel fused-image-based technique that detects OSA using only a single-lead ECG signal. In the proposed approach, a convolutional neural network extracts features automatically from images created with one-minute ECG segments. The proposed network comprises 37 layers, including four residual blocks, a dense layer, a dropout layer, and a soft-max layer. In this study, three time–frequency representations, namely the scalogram, the spectrogram, and the Wigner–Ville distribution, were used to investigate the effectiveness of the fused-image-based approach. We found that blending scalogram and spectrogram images further improved the system’s discriminative characteristics. Seventy ECG recordings from the PhysioNet Apnea-ECG database were used to train and evaluate the proposed model using 10-fold cross validation. The results of this study demonstrated that the proposed classifier can perform OSA detection with an average accuracy, recall, and specificity of 92.4%, 92.3%, and 92.6%, respectively, for the fused spectral images.

## Introduction

Sleep apnea (SA) is one of the most common respiratory disorder and is caused by the complete or partial discontinuation of airflow that accompanies an obstruction of the upper airway for a short time [1, 2]. A complete pause of at least 10 s in the airflow through the upper airway during sleep is usually considered as an apnea-episode.

Three types of SA can be identified, depending on the manner of breathing, namely central SA (CSA), obstructive SA (OSA), and mixed apnea [2]. CSA is caused by instability in the central nervous system and is related to blockage of the airway at the back of the throat. If the airway block is only partially disturbed, then the pathology is termed hypopnea [3]. Such a hypopnea event involves at least 10 s of shallow breathing, which lowers the air volume entering the lungs to below normal levels and causes blood-oxygen desaturation of at least 4%. An occurrence of OSA is recognized when a patient has a complete airflow pause in the upper airway for at least 10 s. Mixed apnea is identified when the apnea begins as a CSA and terminates as an OSA, showing both CSA and OSA characteristics.

Undiagnosed and untreated repetitive apneic episodes can cause a variety of health issues, including excessive daytime sleepiness, cardiovascular and neurological diseases such as memory impairment, high blood pressure, acute coronary syndrome, and congestive heart failure [4, 5]. According to previous studies [2, 6, 7], around 3–7% of adult men and 2–5% of adult women worldwide suffer severely from SA.

In clinical work, the severity of apnea and hypopnea episodes is qualitatively assessed using the apnea–hypopnea index (AHI). The AHI value is defined as the average number of apnea/hypopnea episodes occurring within one hour. In general, a subject showing an AHI value greater than five would be diagnosed with SA [3, 8]. A mild OSA is diagnosed if the AHI value lies between 5 and 15, moderate OSA patients show AHI values between 15 and 30, and severe cases have AHI values above 30 [9].

The most common diagnostic method for OSA suspects is polysomnography (PSG). In PSG, various physiological signals are acquired from sleeping patients, including airflow, respiratory effort, electroencephalogram (EEG), electrocardiogram (ECG), and oxygen saturation (*SaO*_2_). The specific patterns in these physiological signals are then scrutinized by sleep experts to detect sleep-related disorders such as OSA. A PSG study requires dedicated nursing staff and expensive medical equipment specifically designed for PSG data acquisition. The PSG diagnosis method therefore needs dedicated supervision and is time-consuming, expensive, and uncomfortable for patients. Aiming to minimize these technical and economic complications of conventional PSG, several automatic SA detection methods have been proposed during the past two decades. These are usually based on the analysis of the cardiopulmonary (CP) bivariate signal (a combination of heart rate (HR) and respiratory rate (RR) signals), or ECG-derived respiration (EDR).

Respiratory activity is disturbed during an apnea/hypopnea episode, which causes observable variations in the RR signal. Because recording respiratory activity via sensors positioned around the nose is uncomfortable for patients, the RR signal is usually acquired indirectly via EDR signals or inductance plethysmography [10, 11]. The EDR signal is widely used in detecting many sleep-related pathologies because it accurately reflects respiratory activity. Moreover, ECG electrodes can be easily attached to the body without disturbing sleep, unlike direct respiratory sensors.

However, studies have shown that an episode of SA is more strongly associated with signal variability, including heart-rate variability (HRV), morphological variations in the ECG signal [12–14], and variations in the ECG signal’s QRS duration [15, 16]. Therefore, many studies have been carried out based on these observations, and algorithms based on morphological-variation features have tended to show improved performance [13, 17].

Our method also focuses on ECG signal variability, HRV, and morphological variations during an apneic event. It exploits a fused combination of time–frequency representations (TFRs) to intensify the HRV-based and QRS-based variations in ECG signals (scalogram and spectrogram). The presence or absence of apneic events is then detected by means of a deep convolutional neural network (CNN) using fused images created from scalogram and spectrogram representations. To the best of our knowledge, our study is one of few that detects OSA by employing fused spectral images. Compared with other existing methods, our method shows improved performance because it not only combines a variety of TFRs but also exploits recent advances in CNN-based classifiers.

The remainder of this paper is structured as follows. Section 1 summarizes previous work on the detection of OSA-related disorders. Section 2 provides a detailed description of our proposal’s data, method, data preprocessing, image creation, model training, and evaluation criteria. Our results, discussion, and conclusions are presented in Sections 3, 4, and 5, respectively.

### Related work

During the previous two decades, many methods have been proposed to detect SA, using a variety of physiological signals that includes ECG, EDR, and respiratory signals [18–20]. According to Guilleminault *et al*. [21], the occurrence of an apneic event is related to the concomitant variation in the RR intervals in the ECG signal. To date, many studies have considered automatic OSA-detection methods using a single ECG lead. In [22], Khandoker *et al*. proposed an SA-detection method using features extracted from successive wavelet-coefficient of the RR intervals and the EDR signal from the R waves in the QRS complex with a support vector machine (SVM) classifier. In their study, more than 90% of test subjects were classified correctly. Song *et al*. [23] developed a per-segment apnea-detection method using a discriminative hidden Markov model (HMM) based on the ECG signals, where frequency-domain and time-domain features were extracted from the EDR and ECG signals. The per-segment detection accuracy of their model was 86.2% with the PhysioNet Apnea-ECG database.

Kunyang *et al*. [24] proposed a neural-network (NN)-based model that used an HMM for SA classification. In their work, a combination of sparse autoencoders, NNs, and HMMs was used to develop the framework. A classification accuracy of 84.7% was achieved for per-segment apnea detection. Hayano *et al*. [25] proposed a screening method for OSA using cyclic variations in heart rate (CVHR). The agreement between the SA and the presence or absence of CVHR in each one-minute period was found to be 83%. In the study presented in [17], Sharma *et al*. achieved 84.4% accuracy for one-minute ECG signals in detecting apnea using a least-squares (LS) SVM classifier with a Gaussian radial-basis-function (RBF) kernel for features derived from Hermite expansion coefficients. Later, Viswabhargav *et al*. in [8] proposed an apnea detection method whereby EDR and RR signals were utilized to extract sparse residual entropy (SRE) features, using an SVM classifier. In their study, an RBF-kernel-based SVM classifier achieved 85.43% sensitivity and 92.60% specificity for the SRE features.

Tripathy *et al*. [26] introduced a novel method that analyzed the CP signal using fast and adaptive bivariate EMD coupled with cross time-frequency. The CP signal was formulated using both the HR and RR signals derived from the ECG signal. Their method achieved average sensitivity and specificity values of 82.27% and 78.67%, respectively, using an SVM classifier and “random forest” classifiers in a 10-fold cross-validation method.

In [27], Singh *et al*. proposed a method based on the heartbeat interval and EDR, where sliding-mode singular spectrum analysis was used to extract features, with sensitivity and specificity values being 82.45% and 79.72%, respectively.

In these methods, many of the features of the ECG signal used in the classification are derived manually. These include waveform parameters such as instantaneous amplitude (IA) and instantaneous frequency (IF), residual entropy features, statistical features, and other specifically derived features. Some features are derived from the QRS complex and selected manually. In some cases, much manual preprocessing is required when performing specific derivations, including EDR signal extraction and QRS approximation prior to the extraction of specific features. Moreover, most existing approaches use frequency-domain and time-domain representations and nonlinear features derived from physiological signals, where substantial knowledge and relevant experience is required.

To address this issue, Wang *et al*. [28] proposed a method based on a modified LeNet-5 CNN, where feature extraction is automated with an accuracy of 87.6% in the classification of OSA.

Recently, deep learning has become widely implemented in medical imaging and signal analysis because of its advances in pattern recognition and image-based studies. Researchers have also used deep-learning techniques to address ECG-related research issues such as arrhythmia detection [29–34] and other research applications [35, 36]. In these studies, deep neural networks (DNNs) were introduced successfully to extract descriptive and distinguishable features automatically from the input data, which were then used to perform the classification.

McNames *et al*. [37] employed spectrogram signatures calculated from ECGs via fast Fourier Transform (FT) to classify OSA. They obtained a case-based detection accuracy of 92.6%. Singh *et al*. [38] proposed a method based on ECG scalograms that were created via wavelet transforms to detect OSA using a DNN. Their method achieved an accuracy of 86.22% and a sensitivity of 90% in per-minute OSA classification.

In this study, we propose a novel method for OSA detection using fused images created by combining Short-Time Fourier Transform (STFT) and continuous wavelet transform (CWT) representations. A deep CNN model is employed to classify apneic and non-apneic ECG segments using the fused images as inputs. The proposed method does not use any QRS-based features or other manually derived features in performing the classification. Instead of using a limited number of features derived from the QRS complex or other EDR signals, we found that combination of two spectral images carries more discriminative information on the presence or absence of apneic events.

## Materials and methods

### Dataset

To evaluate the proposed method, we utilized the popular and widely used PhysioNet Apnea-ECG database provided by Dr. Thomas Penzel at Philipps University [39, 40]. The dataset comprises single-lead ECG signals from 70 subjects. The recordings are in two groups (a released set and a withheld set), each with 35 subjects. The ECG signals were recorded at a sampling rate of 100 Hz and with 16-bit resolution. Each ECG signal lasted 420–600 min with a mean of 492±32 min. Non-overlapping one-minute ECG segments were annotated as either “OSA” or “Normal,”/but no distinction was made between cases of hypopnea or apnea. The PhysioNet Apnea-ECG database includes both male and female subjects aged from 27 to 63 years with a mean of 43.8±10.8 years. The body weights of the subjects ranges from 53 to 135 kg with a mean of 86.3±22.2 kg. The sleep recordings were obtained from 25 male and 7 female volunteers, including both healthy and OSA subjects [41, 42].

### Method

The TFR of a signal is often used to analyze the information embedded in a variety of signals, including physiological, speech, and geophysical signals. It can be used to identify complex and high-dimensional nonstationary properties of the signal. STFT and CWT are two of the most widely used visual representations for analyzing nonstationary signals. In particular, altered frequencies, amplitudes over time, and morphological variations in ECG signals can be better represented using STFT and CWT instead of FT (see Eqs 1 and 3). The TFR of a signal is often illustrated as a colored image (heat map) in a spectrogram or a scalogram. The spectrogram image usually comprises a visual representation of the STFT, and the scalogram image is a visual representation of the CWT [43].

STFT is used to construct the TFR of the physiological signals as a spectrogram with a constant time–frequency resolution (see Section 2, Eq 1). A constant sliding window along the time axis is employed to create a two-dimensional (2D) representation of the signal at this fixed resolution [41, 44, 45]. As a result of using a constant window, all the frequency information is analyzed at the same time–frequency resolution. In contrast to STFT, the CWT’s wavelet window is scaled and shifted during the transformation. This provides long time windows for low-frequency regions and shorter time windows for high-frequency regions. Therefore, the scalogram provides a more detailed and finer representation of the signal in both low- and high-frequency regions. The mathematical formula for calculating the wavelet coefficients is given in Eq 3. As described in this equation, a basis function, i.e., the mother wavelet *ψ*(*t*), and its scaled and dilated versions are used to decompose the time-domain signal. In both TFRs, the use of a window introduces a compromise between time localization and frequency localization.

In this study, we conducted experiments comparing four types of imaging: scalogram images, spectrogram images, images based on smoothed pseudo Wigner–Ville distribution [46, 47], and fused images (a hybrid version of CWT and STFT images, see Section 2.3), for the identification of apneic events. However, we noted that the Wigner–Ville distribution method has cross-term issues when used with nonstationary signals [48].

Our proposed apnea-detection method is based on deep learning, using a fusion of two spectral images (scalogram and spectrogram images) for one-minute ECG segments (see Fig 4). Each one-dimensional ECG segment in the time domain is transformed into more-detailed 2D forms (scalogram, spectrogram, Wigner–Ville distribution, and fused image), which are used by the CNN to perform image feature extraction and classification.

As explained in the Introduction, the motivation behind combining the two TFRs is to increase the discriminative information in newly formed images. With the Apnea-ECG database providing one-minute-based annotations, the proposed method uses one-minute ECG segments to identify apneic events. Although there are many good reasons for employing TFR or fused TFR in a variety of applications, expert knowledge is expressly needed to extract specific features from such representations. In other words, it is often not realistic to select, analyze, or identify the specific patterns or features in a TFR manually because it contains such fine and complex details. Therefore, utilizing deep learning techniques is the most promising approach to identifying TFR features intelligently and automatically.

**Fig 1:**
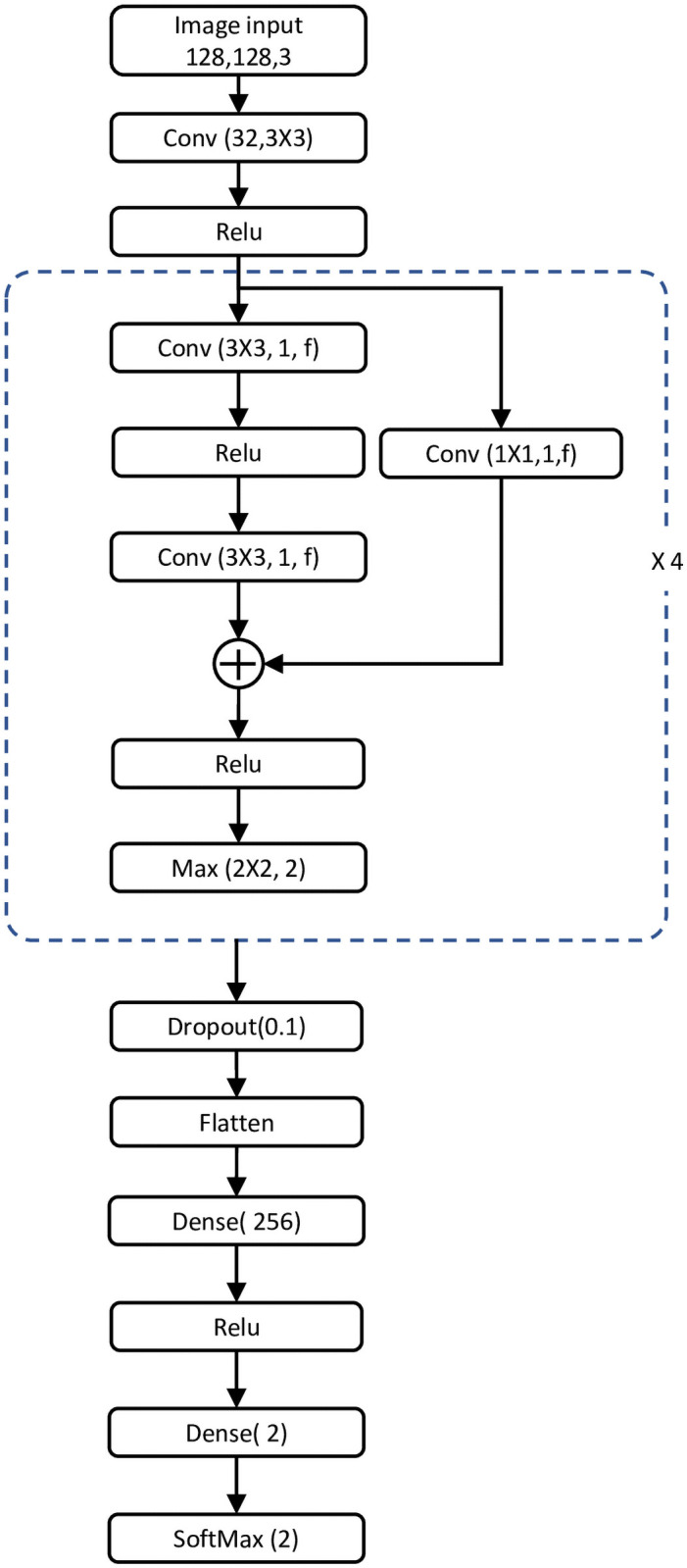
Proposed 2D-CNN network for OSA event detection. *“Conv(k,s,f)”* denotes a convolutional layer where *k*, *s*, and *f* are the kernel size, stride size, and number of filters, respectively. *“Max(p,s)”* denotes a max-pooling layer where *p* and *s* are the pool size and stride size, respectively. The values for the filter sizes *“f”* in the four residual blocks are 32, 64, 96, and 128.

We propose a residual learning approach for performing OSA classification, as shown schematically in Fig 1. A plain CNN is created with stacked layers of linear and nonlinear processing units. These layers enable the network to learn complex and detailed representations at different levels of abstraction [49]. A residual network differs from a plain CNN in having “skip” connections, as shown in Fig 1. Here, activations from the previous layer are reused until the next layer learns its weights. These skip connections help to mitigate gradient vanishing and degradation, which are common problems in large plain networks. Moreover, a residual network can be easily trained to learn a residual mapping with fewer stacked layers than a plain network, with substantially improved performance in image classification [50–52].

### Preprocessing and image creation

**Fig 2:**
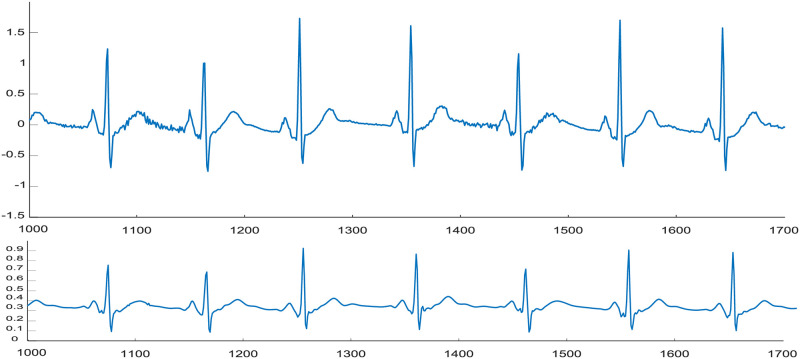
Preprocessing ECG segments. (a) Part of an original ECG segment. (b) The denoised and scaled version.

**Fig 3:**
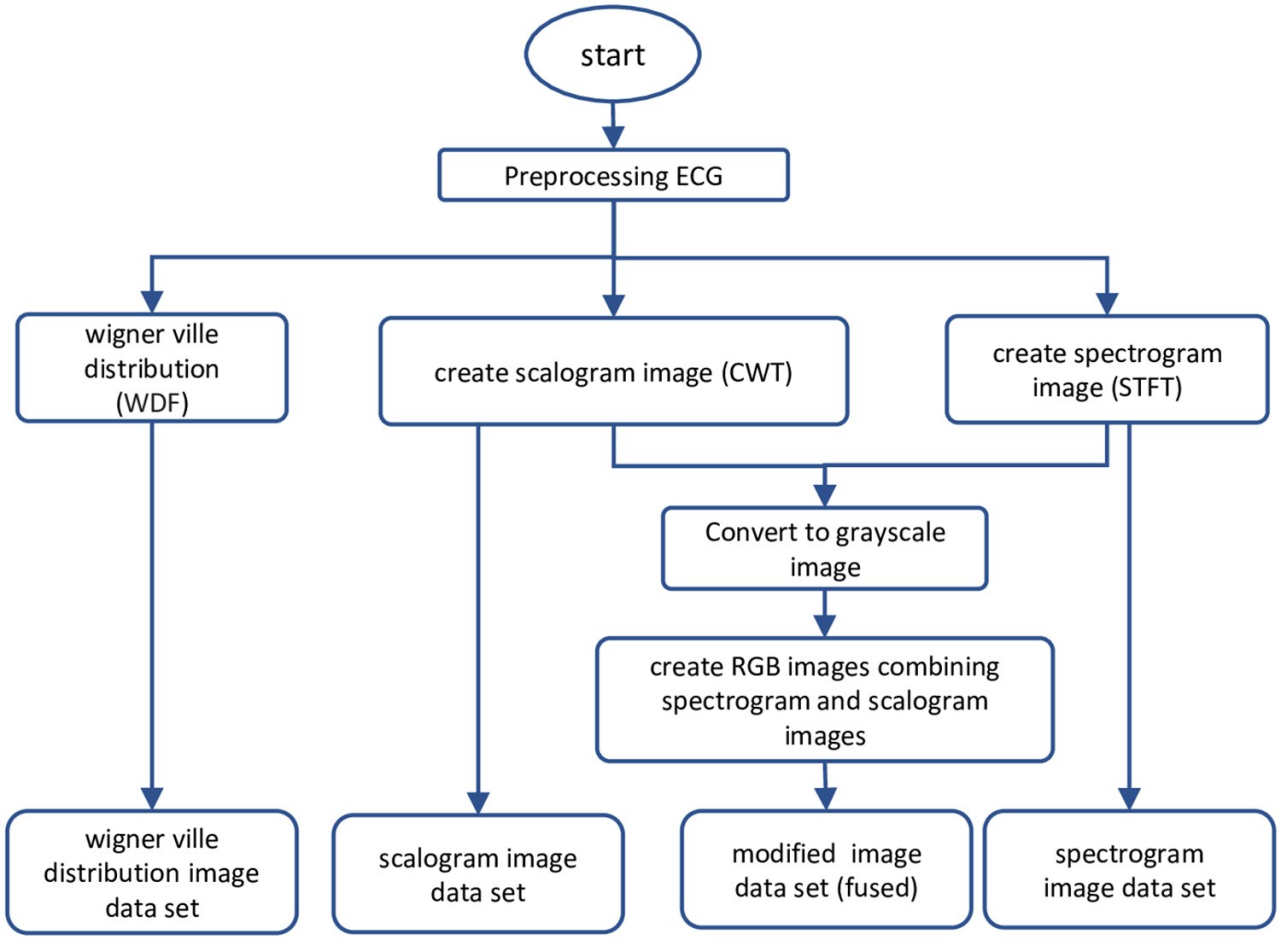
Image dataset creation.

It is usual for a raw ECG signal recorded by a continuous cardiac monitoring process to be corrupted by various types of noise. The presence of noise-related artifacts in the TFR image may cause imprecise estimation of characteristic points and features [53]. Therefore, we performed signal denoising using three of MATLAB’s inbuilt functions: *“wavedec,”* *“waverec,”* and *“cmddenoise”* [46]. First, the raw one-minute segments were transformed into wavelet coefficients using *“wavedec,”* with the *“sym8”* wavelet used to perform the baseline correction. Next, the *“cmddenoise”* function was utilized to perform interval-dependent thresholding for the baseline-corrected signal. Fig 2 shows part of a raw ECG segment and its preprocessed waveform before being transformed into image form. The denoised dataset is transformed into TFRs (four image datasets) as illustrated schematically in Fig 3.

First, we prepared a spectrogram dataset for evaluating the performance of the proposed model. Here, we used MATLAB’s inbuilt function *“spectrogram”* with a *“blackman”* window. While creating the spectrogram images, the window size was set to 64 (640 ms), and the overlap was set to 60 (600 ms) samples [47]. The definition of window function *ω*(*n*) is given in Eq 2. MATLAB’s *“cwt”* function was employed to create the scalogram images using the *“Morse”* analytic wavelet. The scalogram image was formed using the squared modulus of the CWT coefficients as a function of time and frequency, where the frequency is plotted on a logarithmic scale. The height and width of the created scalogram images represent the frequency and time, with the red/green/blue (RGB) colors representing the absolute values of the CWT mapped into a (three-dimensional) color map.

**Fig 4:**
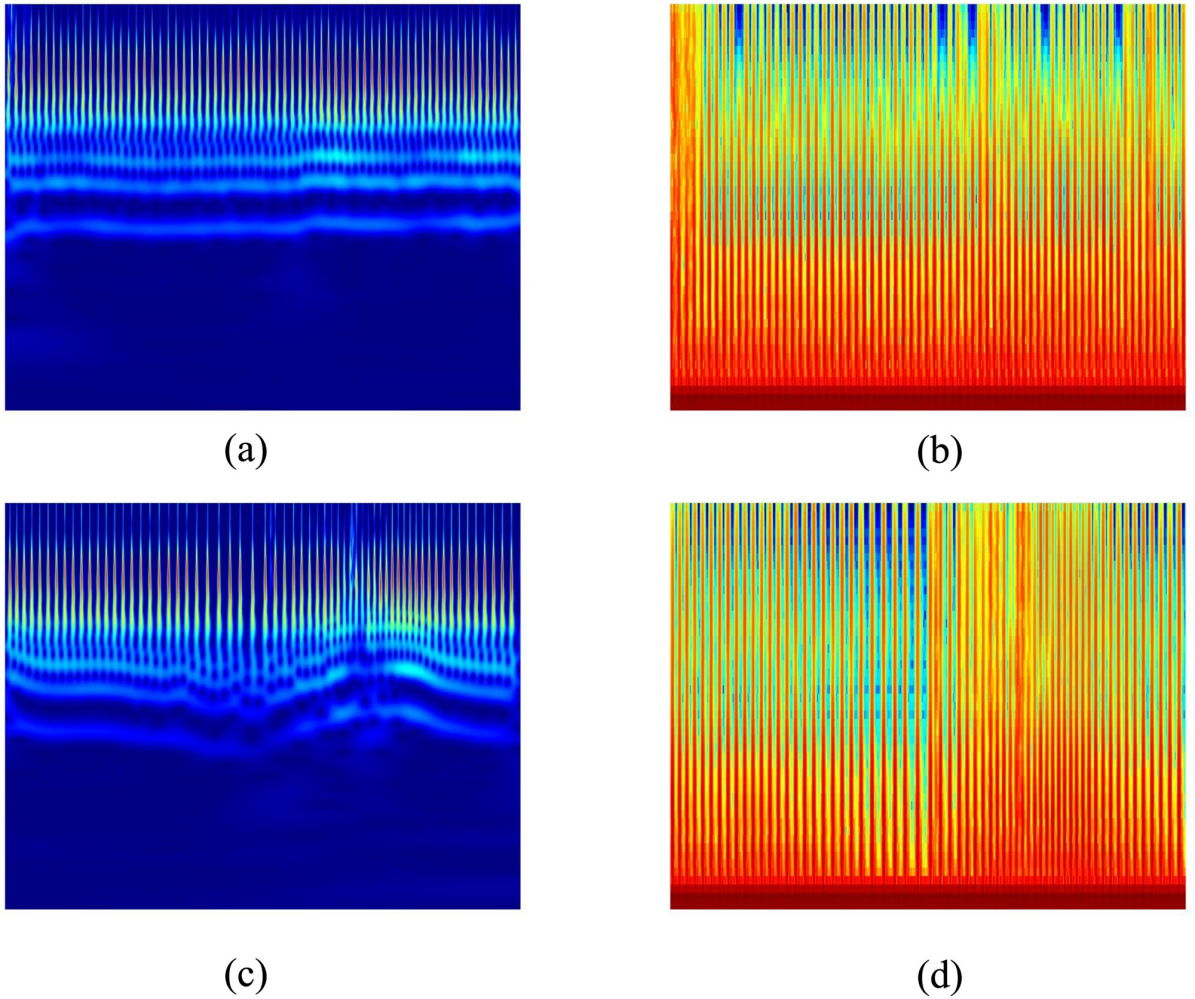
One-minute ECG segments transformed into (128, 128, 3) RGB images. (a) Scalogram image of a normal ECG segment. (b) Spectrogram image of the normal segment. (c) Scalogram image of an apnea ECG segment. (d) Spectrogram image of the apnea segment.

In this study, the *“Morse”* wavelet was used as the mother wavelet for the CWT of the ECG segments, given that it had already been used successfully in many research applications [36, 54, 55]. We saved both sets of images, generated with *“cwt”* and *“spectrogram,”* using the *“gcf”* command. Fig 4 shows both types of TFR image created for normal and apneic ECG segments.

For comparison purposes, we also used MATLAB to prepare a TFR involving a smoothed pseudo Wigner–Ville distribution, with time and frequency windows used for the smoothing.

$$\begin{array}{c} \;X_{STFT}[m,n]=\;\sum_{k\;=\;0}^{L-1}x[k]\omega [k-m]e^{-j2\pi nk/L}, \end{array}$$ (1)

$$\begin{array}{c} \omega (n)=0.42-0.5cos(\frac{2\pi n}{L-1})+0.08cos(\frac{4\pi n}{L-1}), \end{array}$$ (2)

where *L* is the window length, *x*[*k*] is the input ECG signal, and *ω* is the window function. The log values of *X*_*STFT*_[*m*, *n*] are used to create the RGB color image (the spectrogram image) [44].

$$\begin{array}{c} W_{x}(s,\tau )=\frac{1}{\sqrt{s}}\int_{-\infty }^{+\infty }x(t)\psi^{*}(\frac{t-\tau }{s})dt, \end{array}$$ (3)

where *W*_*x*_(*s*, *τ*) is the wavelet coefficient, *x*(*t*) is the ECG signal, *ψ*(*t*) is the basis function (mother wavelet) conjugate, *s* is the scale, and *τ* is the time parameter.

In creating spectral images, we pretested several window sizes, overlap lengths, windows, and other parameters, using a small amount of randomly selected data to make sure that appropriate and comprehensible images were generated, based on visual inspection and CNN performance.

**Fig 5:**
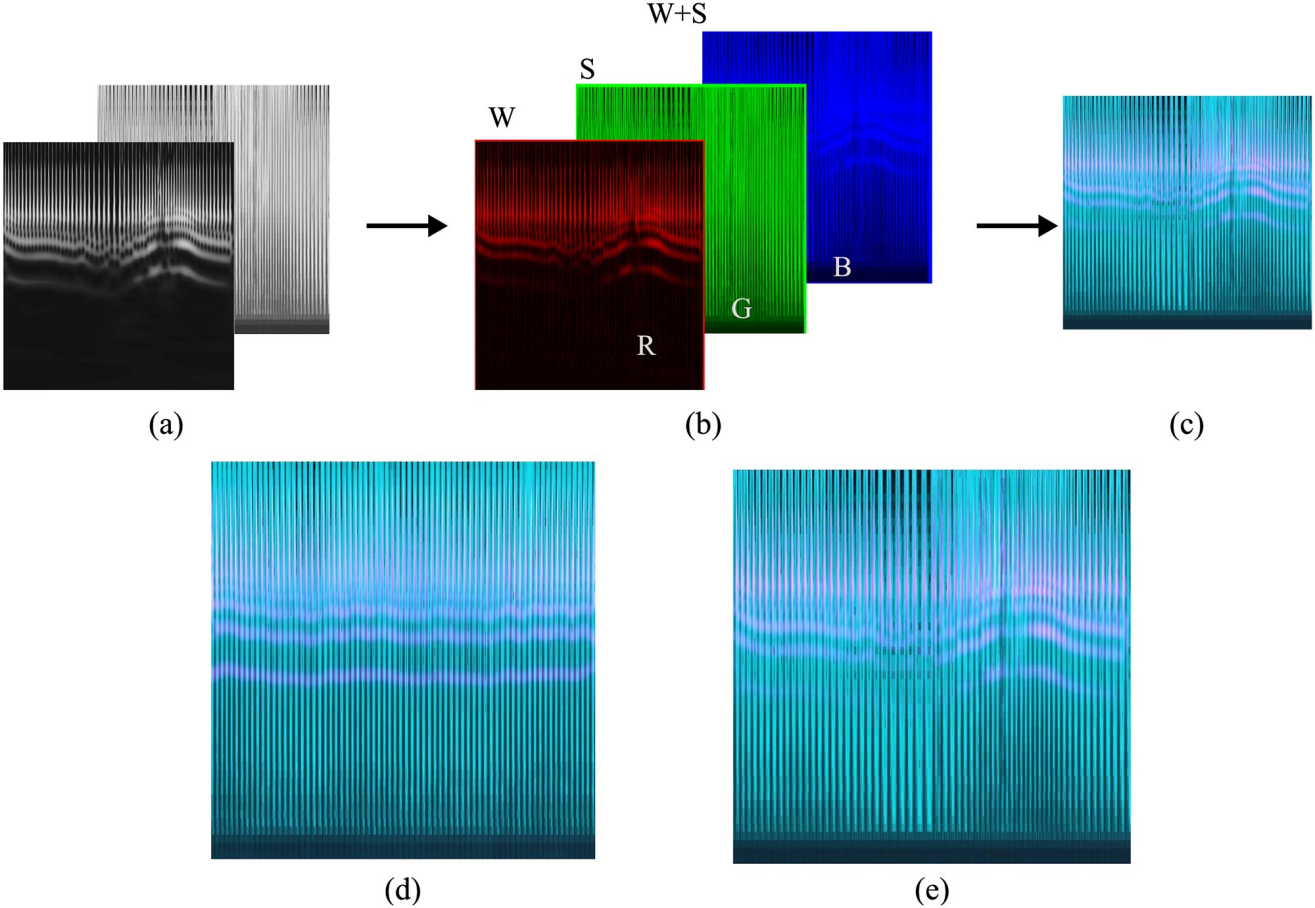
Fusing the scalogram and spectrogram for an apnea ECG segment. (a) Gray-scaled scalogram and spectrogram images. (b) RGB components of the modified image. (c) Fused image. (d) Fused image of a normal ECG segment. (e) Fused image of an apnea ECG segment.

After confirming the most appropriate parameters for image creation, the scalogram, spectrogram, and pseudo Wigner–Ville distribution image datasets were constructed. Finally, the fused image dataset was generated using the scalogram and spectrogram images as shown in Fig 5. Fused images were created by embedding gray-scale values of the scalogram and its matching spectrogram into three layers of an RGB image. To embed the CWT and STFT representations into one image, the gray-scale values of the scalogram image were used as the “red” component of the new image, and the “green” component was formed using the corresponding gray-scale values of the spectrogram. The “blue” layer was created by the addition of the gray-scale values of the scalogram and spectrogram. In this way, the three RGB layers of the fused image accommodated picture elements from both scalogram and spectrogram images. As shown in Fig 5, the modified image is therefore a hybrid version of the CWT and STFT images that carries more discriminative information than does the original form. In other words, each pixel or point represents the spectral presence of the ECG wave derived from both TFRs.

### Proposed model

The proposed CNN architecture is shown in Fig 1, where the CNN comprises four residual blocks sharing the same architecture but with different hyperparameters. The CNN has 37 layers, including the convolutional, max-pooling, dense, and other layers. Overall, there are 13 convolutional layers and four max-pooling layers. The model starts with 32 convolutional filters (3 × 3) followed by a rectified linear (ReLU) activation layer. The output is then passed to a series of residual blocks, as shown in Fig 1. Each residual block is formed as two consecutive convolutional layers and a skip connection through a 1 × 1 convolutional layer to restore the dimensionality. After the addition layer in each residual block, a ReLU activation layer and a 2 × 2 pooling layer with a stride size of two are utilized to summarize the feature map generated by each residual block. The number of filters (*“f”* in Fig 1) used in all convolutional layers in the same residual block is kept unchanged. The max-pooled output of each residual block is passed to the next residual block. The number of filters in a residual block is successively increased (32, 64, 96, and 128). The max-pooled output of the last residual block is then passed to a 0.1 dropout layer to avoid model overfitting. Adding a dropout layer is a recognized training technique whereby some nodes are dropped out randomly during training. This is a very effective regularization method, limiting overfitting and reducing the generalization error in a DNN model.

Finally, the flattened output of the dropout layer is passed to a fully connected layer with 256 units followed by *“ReLU”* activation. The classification layer is a soft-max layer, where the output of the network is normalized to a probability of *y*_*k*_, as specified by Eq 4. The fully connected dense layer with 256 units followed by *“ReLU”* activation works as the classifier for the features derived from the deep stacked residual blocks.

$$\begin{array}{c} y_{k}=\frac{\exp\{a_{k}\}}{\sum_{j=1}^{K}\exp\{a_{j}\}},\;\;\;\;\;k=1,2, \end{array}$$ (4)

where *a*_*k*_ is the activation (a linear weighted sum of the hidden nodes) of the *k*^*th*^ neuron in the soft-max layer, and *y*_*k*_ is the probability of the individual class.

### Implementation of model training

**Fig 6:**
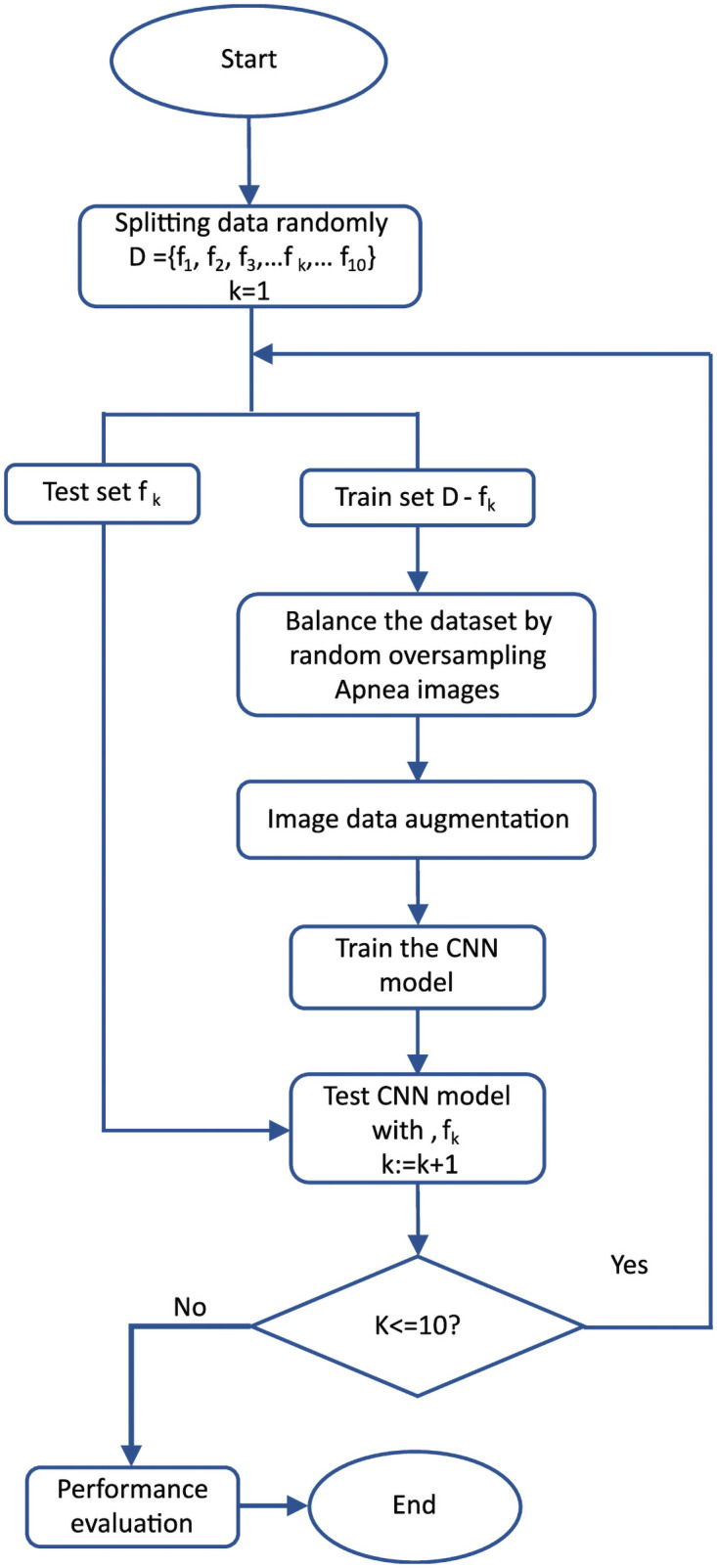
Schematic diagram of the training procedure for the proposed 2D-CNN model with 10-fold cross validation.

The proposed model was implemented and trained using the MATLAB R2020a deep-learning toolbox [56]. The model was trained with graphics processing unit support (NVIDIA GEFORCE GTX 1070) using 10-fold cross validation [57]. We selected 20,000 “normal” ECG segments and 13,062 “OSA” ECG segments to train the proposed network. The image data (*D*) were randomly split into 10 equal subsets {*f*_1_, *f*_2_, *f*_3_, …*f*_*k*_, …, *f*_10_}, with one subset chosen as the test dataset and the remainder used to train the model, resulting in one model for each of the 10 folds (see Fig 6).

After determining the test and training datasets, random oversampling was performed to balance the dataset and prevent the model from being overfitted. Here, the training set comprised 18,000 normal one-minute ECG segments for each fold. To balance the dataset, we randomly copied apnea images (the minority class) so that the total number of OSA images was also 18,000. All training images, including the randomly oversampled images, were then subjected to fold-wise image augmentation using random rotation (–8 to +8 degrees), random horizontal translation (–30 to +30 pixels), vertical translation (–10 to +10 pixels), random shearing (–5 to +5 degrees), and random horizontal flipping. Small-scale augmentation was used because spectral images tend to be consistent and steady compared with normal still images taken by a camera, where high sample variation can occur, including large rotations and scaling, vivid colors, and special effects. The full training procedure is shown in Fig 6. In this procedure, the mini-batch size was 128, and the model was evaluated after every 256 iterations both to ensure that the model continued towards convergence during training and to visualize the training process (see Fig 10).

A back-propagation algorithm was used to train the whole model by optimizing the cross-entropy error *E*_ce_ between the predicted classes and the actual class, as specified in Eq 5, using the *“Adam”* optimizer with an initial learning rate of 0.001, as suggested in [58, 59]. Each fold was run for up to 48 epochs, until the training loss between consecutive batch updates ceased to improve. After training all models, the best model for each fold was selected for evaluation, according to its validation accuracy.

$$\begin{array}{c} E_{\text{ce}}=-\sum_{n}\sum_{k=1}^{K}t_{n,k}\log y_{n,k}, \end{array}$$ (5)

where *y*_*n*,*k*_ is the actual output of node *k*, *n* is the number of examples in the mini-batch, and *t*_*n*,*k*_ *ϵ*{0, 1} are the target outputs [60].

### Performance evaluation

To evaluate the proposed model, we used overall accuracy, per-class recall (RE), per-class precision (PR), per-class specificity (SP), and per-class *F*_1_ score (F1) as defined in Eqs (6–10), respectively [61]. As described in the Method section, the average result of the 10 folds for each performance metric was calculated to reflect the final performance of the proposed CNN.

$$\begin{array}{c} \text{Accuracy}=\frac{TP+TN}{TP+TN+FP+FN} \end{array}$$ (6)

RE (also known as the probability of detection, true positive rate, or sensitivity) reflects the correctly predicted proportion of all positive samples.

$$\begin{array}{c} \text{RE}=\frac{TP}{TP+FN} \end{array}$$ (7)

PR (also known as the positive predictive value) reflects the proportion of positive predictions that are actually correct.

$$\begin{array}{c} \text{PR}=\frac{TP}{TP+FP} \end{array}$$ (8)

SP (also known as true negative rate) reflects the proportion of negatives that are correctly detected.

$$\begin{array}{c} \text{SP}=\frac{TN}{TN+FP} \end{array}$$ (9)

The F1 score denotes the harmonic mean of PR and RE, which considers both metrics to give an optimal measure for analyzing model performance.

$$\begin{array}{c} F1=2\cdot \frac{\text{precision}*\text{recall}}{\text{precision}+\text{recall}} \end{array}$$ (10)

where TP, TN, FP, and FN denote true positives, true negatives, false positives, and false negatives, respectively.

## Results

In this study, an image-based method for OSA detection in one-minute ECG segments is proposed. To investigate the effectiveness of the proposed method, we compared it with other existing methods. Because we used all subjects in training the network using a 10-fold cross-validation method, we compared only the per-segment OSA-detection performance. It should be noted that the test dataset was isolated from the training data before performing random oversampling and was not subjected to image augmentation.

**Fig 7:**
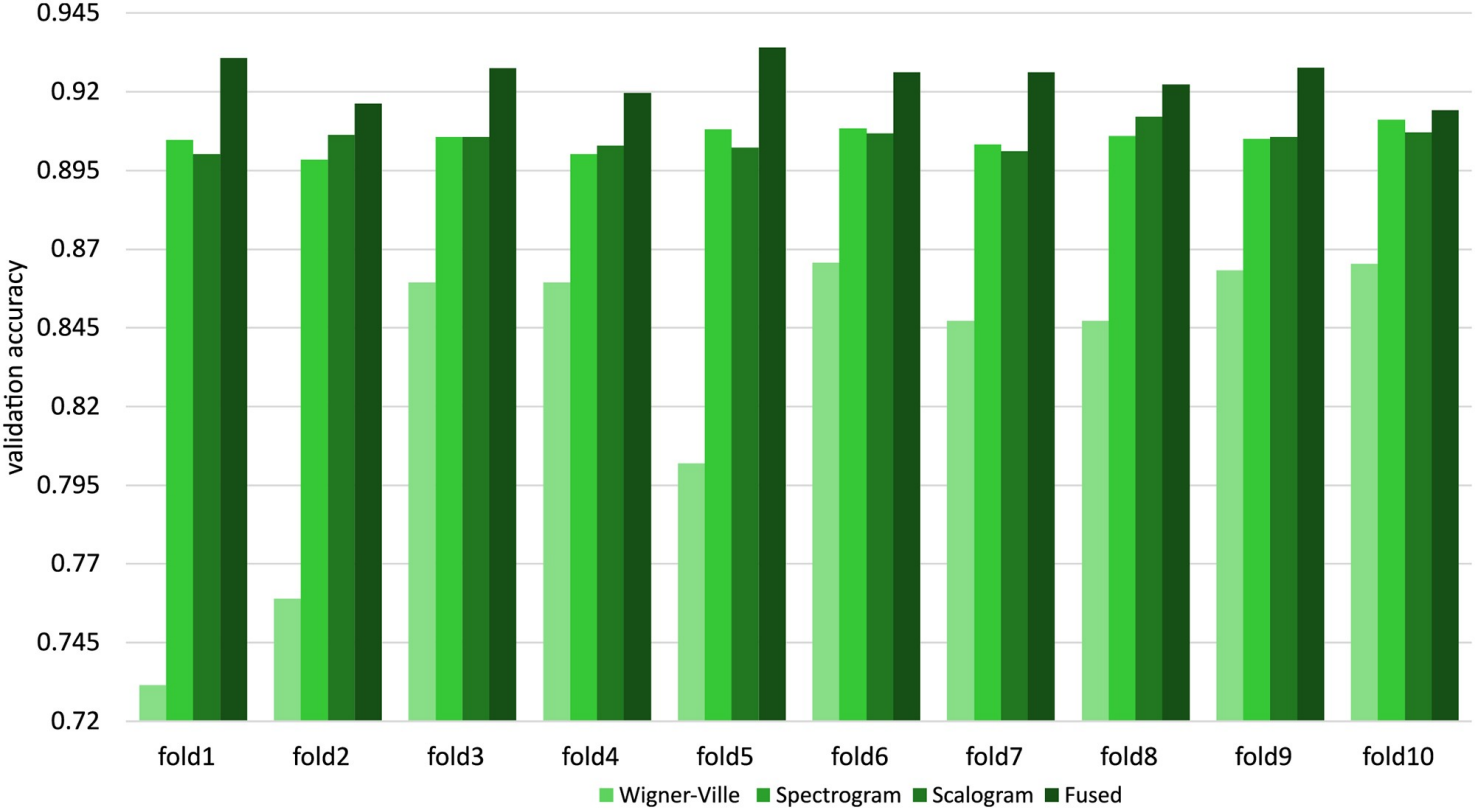
Distributions of validation accuracy for TFR images and fused images over 10 folds.

**Fig 8:**
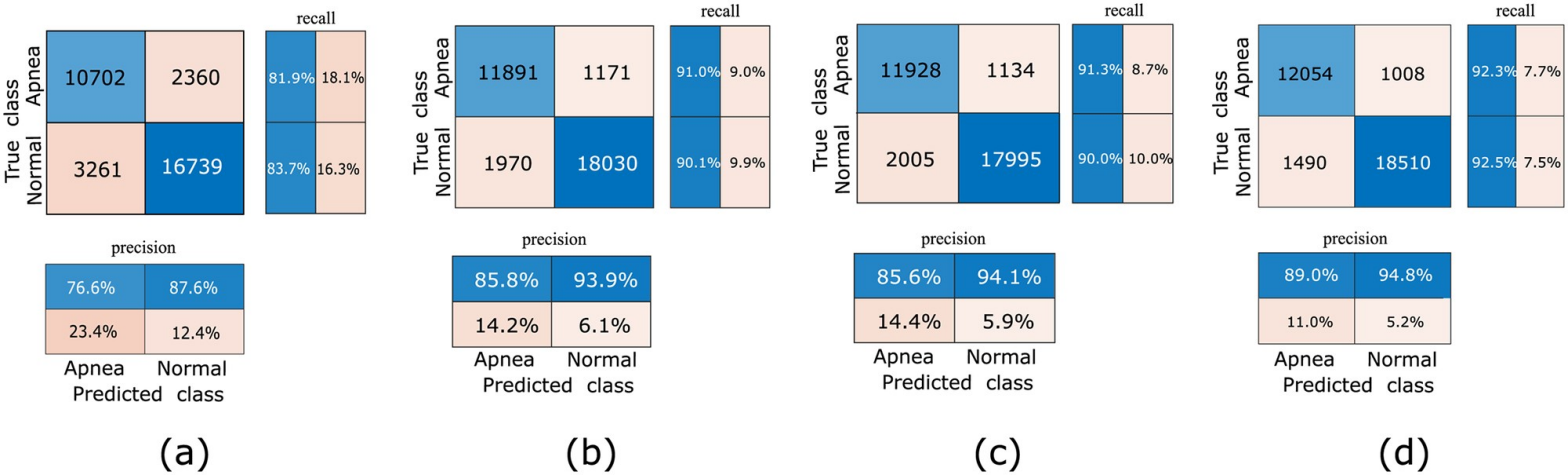
Confusion matrices for per-segment apnea detection, with classwise PR and RE shown in the bottom and right-hand boxes, respectively: (a) Wigner–Ville distribution images, (b) scalogram images, (c) spectrogram images, and (d) fused images.

**Fig 9:**
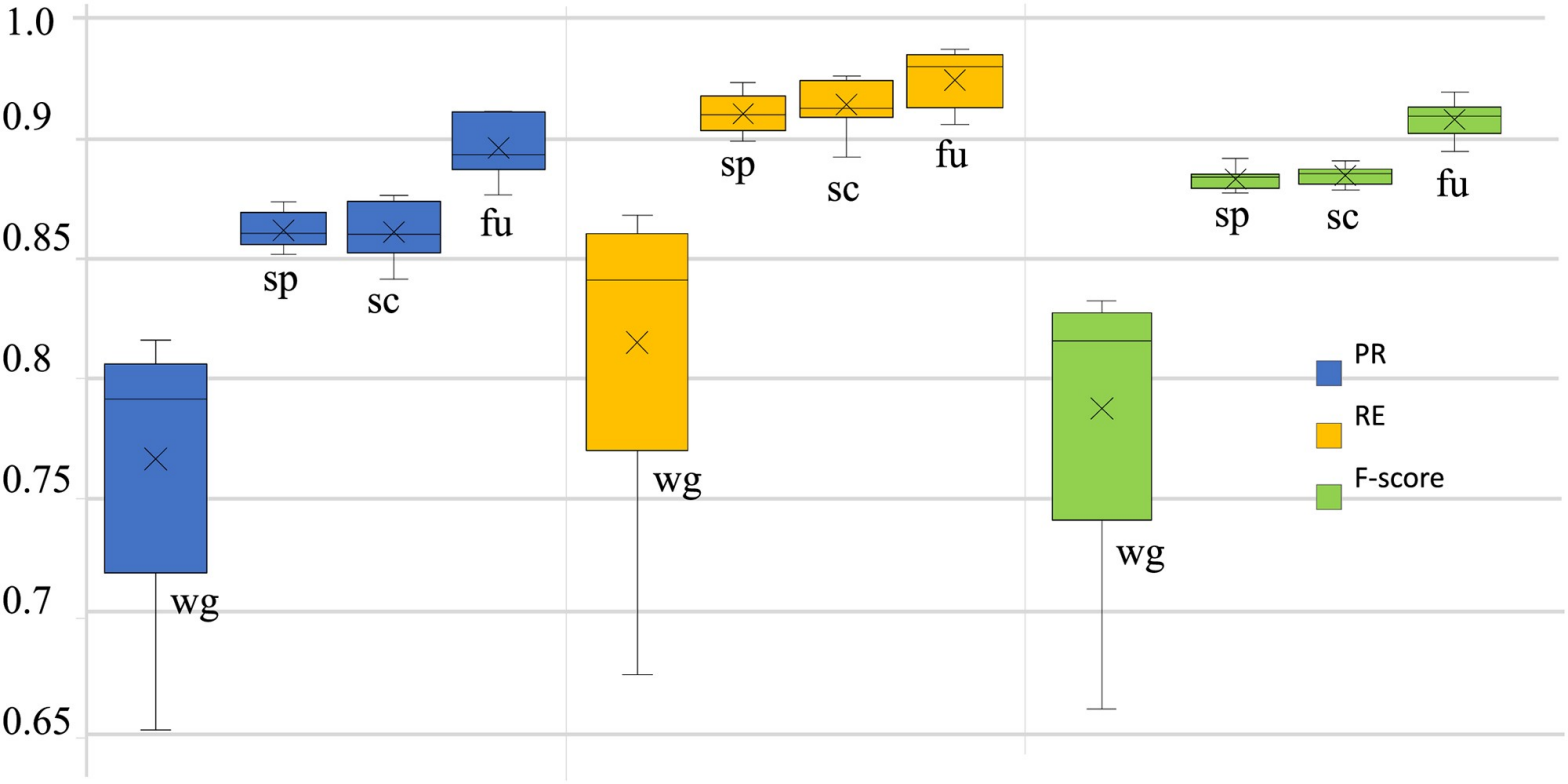
IQR plots of PR, RE, and F1 for apnea detection obtained across all folds. The center line indicates the median, the box limits indicate the upper and lower quartiles, the whiskers indicate 1.5 × IQR, and × indicates the mean. The images are Wigner–Ville distribution images (wg), scalogram images (sc), spectrogram images (sp), or fused images (fu).

Figs 7 and 8 show the validation accuracies for each fold during the 10-fold cross validation and the confusion matrices, respectively. Fig 9 shows interquartile range (IQR) plots for performance metrics calculated across all folds.

Table 1 shows the overall macro average of performance metrics for the proposed model with 10-fold cross validation. The table shows that the performance measures are very similar for all image types other than the Wigner–Ville distribution images.

**Table 1:** Overall performance in per-segment apnea detection TFR images and fused images.

	Acc%	PR%	RE%	SP%	F1%
Wigner distribution	82.9	76.6	81.9	83.7	79.2
Scalogram images	90.5	85.8	91.0	90.2	88.3
Spectrogram images	90.5	85.6	91.3	90.0	88.4
Fused images	92.4	89.0	92.3	92.6	90.6

When considering the validation accuracy for all folds, as shown in Fig 7, there is no great variation between folds, which indicates that the model can be generalizable to other datasets.

As shown in Fig 9, the other performance metrics, including the *F*_1_ score, also show very small variation across the folds. Although the means of the performance metrics are nearly identical for scalogram and spectrogram images, the performance metrics show slightly higher variability across the folds in the scalogram case. The overall accuracy and F1 scores for per-segment OSA detection with scalogram images are 90.5% and 88.3%, respectively. The same measures for the spectrogram dataset are 90.5% and 88.4%. However, the proposed CNN model achieves the highest performance for the fused images, achieving 92.4% overall accuracy and a 90.6% F1 score. The variability of all measures for the fused images is slightly higher than for the corresponding scalograms and spectrograms. The lowest performance occurs in all cases for the Wigner–Ville distribution images, with the greatest variation in performance metrics across the folds.

**Fig 10:**
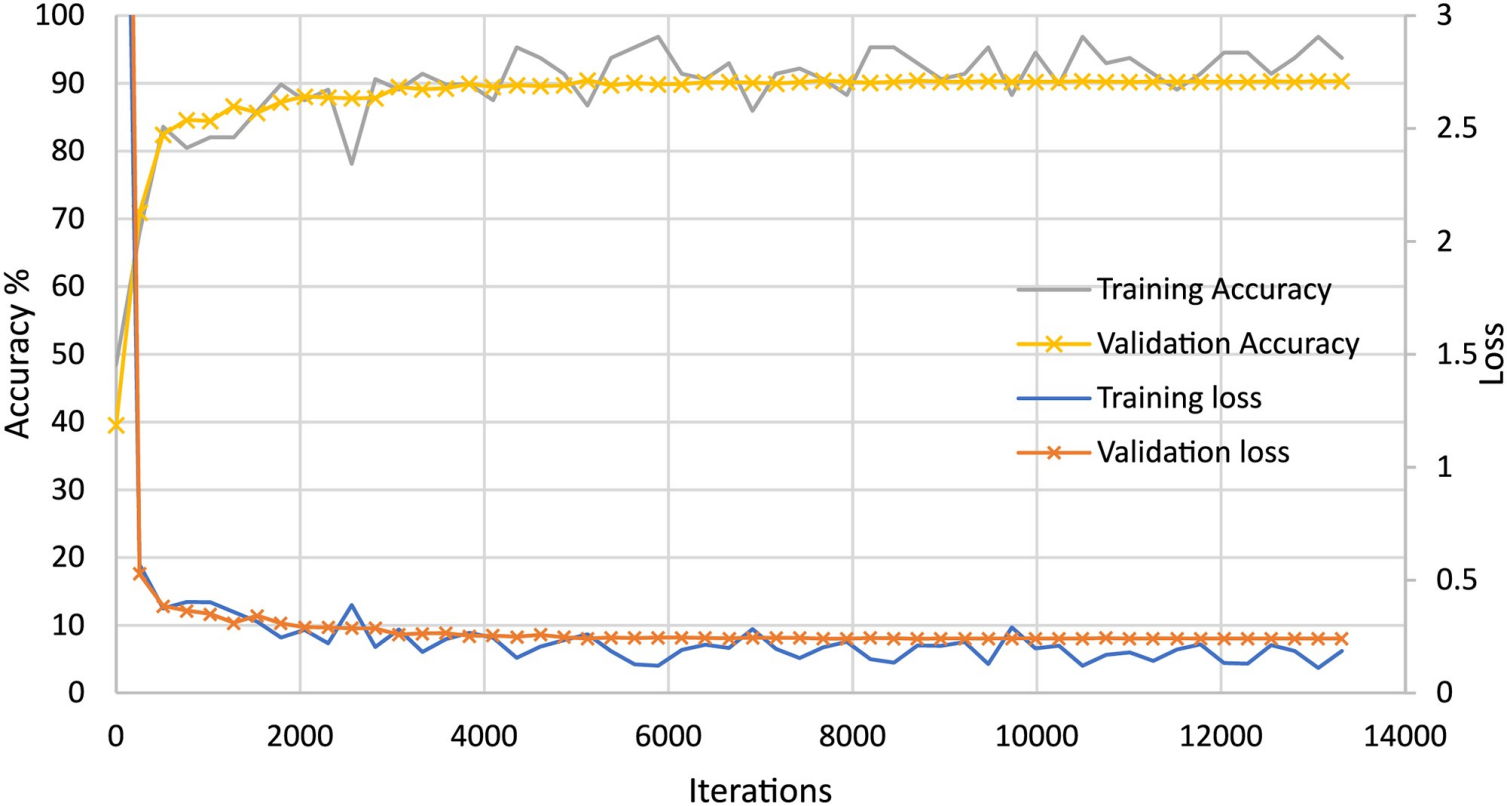
Accuracy-loss graph of the proposed CNN (for the lowest-performing model).

Fig 10 shows an accuracy loss plot for the weakest classifier of fused images. The learning curves confirm that the parameters selected for image creation and the CNN are appropriate for discriminating between OSA and normal ECG segments.

### Robustness evaluation

**Fig 11:**
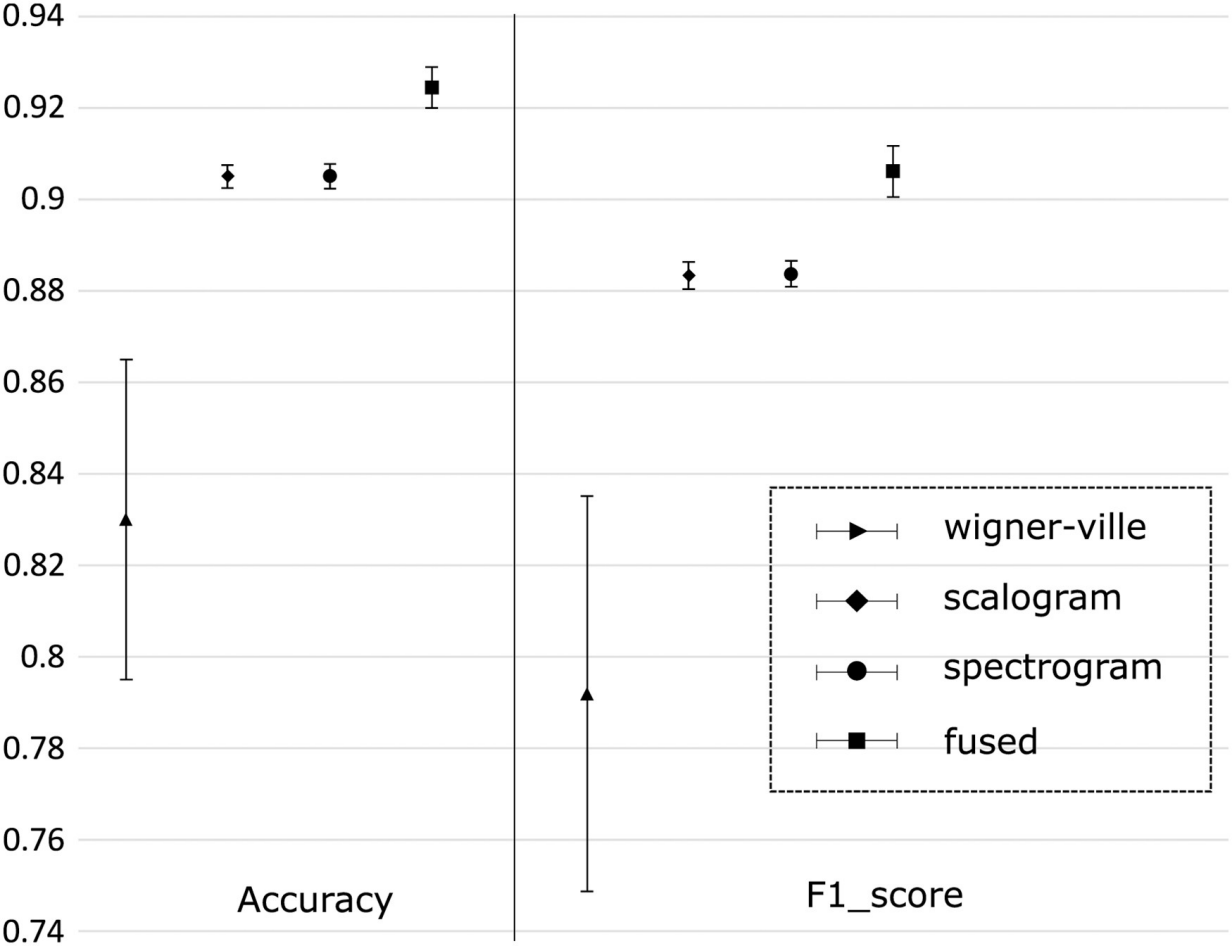
Overall 10-fold cross-validation results for per-segment apnea detection with Wigner–Ville distribution, scalogram, spectrogram, and fused images. Black lines indicate the corresponding 95% confidence interval.

The PhysioNet Apnea-ECG database is a relatively small dataset, with the withheld dataset and the training dataset containing 35 recordings each. Therefore, using a single withheld dataset for validation might be unfair, given that we are training a deep-learning model, which requires more data than the other machine-learning methods. To address this, we used 10-fold cross validation to test the robustness of the proposed CNN with the entire dataset (70 recordings), which was randomly divided into 10 subsets, as shown in Fig 6. Fig 11 shows the average accuracy and F1 score for per-segment OSA detection, with their 95% confidence intervals (CIs) calculated for 10 cross-validation steps. According to Fig 11, the proposed CNN model demonstrates consistent performance for all image types (excluding the Wigner–Ville distribution) in terms of validation accuracy and F1 score with a small 95% CI. The model obtained accuracies of 90.5±0.3%, 90.5±0.3%, and 92.4±0.5% for per-segment OSA detection using scalogram, spectrogram, and fused images, respectively. Similarly, the F1 scores were 88.3±0.3%, 88.4±0.3%, and 90.6±0.6%, for the scalogram, spectrogram, and fused images, respectively. For the pseudo Wigner–Ville images, significantly weaker performance (accuracy = 82.99±3.49% and F1 score = 79.19±7.31%) is observed because the Wigner–Ville distribution method has cross-term issues.

### Comparison with existing methods

Because the PhysioNet Apnea-ECG database has been available for some time, several automatic OSA-detection approaches exist in the literature. Here, we compare our method with those that also used the PhysioNet Apnea-ECG database. Note that we do not consider per-recording detection performance because we trained our network via 10-fold cross validation after aggregating the entire data set.

Table 2 summarizes the performance of the proposed CNN method relative to other existing methods, with respect to per-segment OSA detection. As shown in the table, our method achieved the best performance in terms of overall accuracy, sensitivity, and specificity. In particular, we can compare our method with that of Tao Wang *et al*. [62]. Their method obtained an overall accuracy, sensitivity, and specificity of 87.3%, 85.1%, and 88.7%, respectively for the withheld dataset, whereas our method achieved an average accuracy, sensitivity, and specificity of 92.4%, 92.3%, and 92.6%, respectively, representing a significant performance improvement with the same dataset. Furthermore considering the robustness evaluation, we improved the accuracy by ≈6%, with a smaller CI (≈±0.45%) (for 10-fold cross validation) than their ±1.5% evaluated using 7-fold cross validation for the entire dataset.

**Table 2:** Performance comparison of proposed and previous methods for per-segment apnea detection.

Reference	Method	Validation	Acc (%)	Sen(%)	Spe(%)
Viswabhargav *et al*. (2019) [8]	SRE features with Fourier dictionaries (SVM)	10-fold (SVM)	78.1	78.0	78.1
		subject-specific (SVM)	-	85.4	92.6
Tripathy *et al*.(2020) [26]	Cardio-pulmonary signal/ fast and adaptive bivariate EMD coupled with cross time-frequency analysis	10-fold (SVM)	-	73.2	73.1
		subject-specific (SVM)	-	82.3	78.7
Singh *et al*.(2020) [27]	Instantaneous amplitude and instantaneous frequency-based features/ EDR and HBI signals	10-fold (SVM-RBF)	-	82.4	79.7
		leave-one-out(DNN)	-	63.5	79.9
Sharma *et al*.(2016) [17]	Hermite basis functions	10-fold LS-SVM (RBF Kernel)	83.8	79.5	88.4
Song *et al*.(2016) [23]	HMM–SVM	withheld dataset	86.2	82.6	88.4
Varon *et al*.(2015) [5]	LS-SVM	fixed-size method	84.7	84.7	84.7
Li *et al*.(2018) [24]	EDR signal NN and HMM	withheld dataset	84.7	88.9	82.1
Singh *et al*.(2019) [38]	scalogram images (Morlet wavelet), DNN and Decision fusion	withheld dataset	86.2	90	83.8
Wang *et al*.(2019) [62]	RR interval and R-peak based features, time window and ANN	withheld dataset/7-fold	87.3	85.1	88.7
Proposed method	Smoothed pseudo Wigner–Ville, CNN	10-fold	82.9	81.9	83.7
	Scalogram images (Morse wavelet), CNN		90.5	91.04	90.2
	Spectrogram images, CNN		90.51	91.32	89.98
	Fused images, CNN		92.4	92.3	92.6

In addition, when compared with most previous methods [5, 17, 23, 24] using the same dataset, our method has the best apnea-detection confidence, as shown in Table 2 for all performance metrics. For example, Singh *et al*. [38] proposed an image-based OSA-detection method that used a CNN model based on AlexNet. This method obtained a validating accuracy of 86.2%, a sensitivity of 90%, and a specificity of 83.8% using scalogram images (227, 227, 3). Although their model showed comparatively good sensitivity, our model achieved much better performance in terms of validation accuracy and specificity in addition to sensitivity. Their prediction model is a plain DNN model with five convolutional layers compared with our better-performing model that contains four residual blocks.

Although other recent approaches [8, 17, 26, 27] have performed well for this dataset, our approach has demonstrated better performance metrics because the proposed method adopts recent advances in deep learning that enable the most appropriate features to be extracted automatically. In these other studies, specific features are derived via the ECG or EDR signal, forcing the classifier to depend on manually derived features. However, it should be noted that deep learning models require balanced datasets for optimal performance, unlike other models. Another possible drawback of the proposed method is that it requires conversion of the time-domain signal into two separate TFRs before making its predictions.

## Discussion

The proposed CNN performs well because it is trained using the entire dataset with 10-fold cross validation, which avoids overfitting and provides greater sample variation in the training. In addition, we randomly oversample the apnea images and perform image augmentation, which balances the dataset and improves the number of examples by creating modified versions of the images, including the fused images. The augmented training dataset helps to create skillful models and improves the ability to generalize the model for unseen data. Most importantly, the fused images we used in our study provide a satisfactory blend of discriminative features, where both CWT and STFT features are hybridized.

In contrast to other methods, note that our model detects OSA segments without segmenting the QRS complexes in ECG signals, which provides a robust approach to OSA detection. Moreover, the model can be adapted to predict apneic events using arbitrarily long ECG segments (e.g., 10 s or 20 s) because we use RGB images that can be generated for any ECG-segment length and resized to (128, 128, 3) for input to the model.

## Conclusions

The purpose of this study is to describe the implementation of a robust automatic OSA-detection method based on fused TFR images. Our CNN model for per-segment OSA detection detects OSA events using images corresponding to one-minute ECG segments in any of the Wigner–Ville distribution, scalogram, spectrogram, or fused-image formats. The results for accuracy and other performance metrics demonstrate that our model not only labels apneic events automatically but also outperforms existing methods for automatic OSA detection. Our model achieved an overall accuracy of 92.4% for fused images created from scalogram and spectrogram images. Another important aspect of this work is that the model can be used with arbitrary ECG-segment lengths because the ECG segments are converted to RGB images before input into the prediction model. Moreover, no manual feature extraction is required, which depends on the experience and specific domain knowledge of the researchers. Because our model is based on a single-lead ECG channel, it could be used in wearable electronics or a smart home-monitoring systems. This would be cheaper and more convenient than having to use a conventional sleep-study environment. However, our method has some limitations. Because the PhysioNet Apnea-ECG database provides only two types of annotation (apnea and normal), the proposed model cannot classify apnea subtypes (e.g., hypopnea). In future work, we aim to extend our model to detect these different types of apnea. In addition, we will consider modifying the proposed approach using multiple apnea datasets and will investigate different fusing techniques to improve the performance.

## Supporting information

S1 File (PDF)
