# Supplementary material for: A fused-image-based approach to detect obstructive sleep apnea using a single-lead ECG and a 2D convolutional neural network
Source: PLoS One. 2021 Apr 26;16(4):e0250618. doi: 10.1371/journal.pone.0250618 (PMC8075238; doi:10.1371/journal.pone.0250618)
Supplement: S1 File — (PDF) [file pone.0250618.s001.pdf]

# S1 File

*S1 Table. Sleep Apnea data set*

| Record | Length | non-apn minutes | apnea minutes | hours w/apnea | AI (the number of apneas observed per hour) | HI (the number of hypopneas observed per hour) | AHI (sum of AI and HI) | Age | Sex | height (cm) | weight (kg) |
|---|---|---|---|---|---|---|---|---|---|---|---|
| **a01** | 490 | 20 | 470 | 9 | 12.5 | 57.1 | 69.6 | 51 | M | 175 | 102 |
| **a02** | 529 | 109 | 420 | 9 | 57.2 | 12.3 | 69.5 | 38 | M | 180 | 120 |
| **a03** | 520 | 274 | 246 | 9 | 38.4 | 0.7 | 39.1 | 54 | M | 168 | 80 |
| **a04** | 493 | 40 | 453 | 9 | 73.4 | 4 | 77.4 | 52 | M | 173 | 121 |
| **a05** | 455 | 179 | 276 | 8 | 35 | 6 | 41 | 58 | M | 176 | 78 |
| **a06** | 511 | 305 | 206 | 8 | 16.6 | 8.1 | 24.7 | 63 | M | 179 | 104 |
| **a07** | 512 | 190 | 322 | 9 | 46 | 17 | 63 | 44 | M | 177 | 105 |
| **a08** | 502 | 313 | 189 | 7 | 32 | 10 | 42 | 51 | M | 179 | 88 |
| **a09** | 496 | 115 | 381 | 9 | 23.1 | 8.6 | 31.7 | 52 | M | 178 | 82 |
| **a10** | 518 | 418 | 100 | 6 | 11 | 10 | 21 | 58 | M | 176 | 78 |
| **a11** | 467 | 245 | 222 | 8 | 11 | 3 | 14 | 58 | M | 168 | 103 |
| **a12** | 578 | 44 | 534 | 10 | 70 | 10.2 | 80.2 | 52 | M | 173 | 121 |
| **a13** | 496 | 252 | 244 | 9 | 32 | 10 | 42 | 51 | M | 179 | 88 |
| **a14** | 510 | 127 | 383 | 8 | 17.3 | 37.4 | 54.7 | 51 | M | 175 | 102 |
| **a15** | 511 | 143 | 368 | 9 | 46 | 6 | 52 | 60 | M | 176 | 113 |
| **a16** | 483 | 163 | 320 | 7 | 17 | 24 | 41 | 44 | M | 177 | 105 |
| **a17** | 486 | 328 | 158 | 5 | 21 | 12 | 33 | 40 | M | 179 | 96 |
| **a18** | 490 | 52 | 438 | 9 | 75.5 | 6.9 | 82.4 | 52 | M | 178 | 82 |
| **a19** | 503 | 298 | 205 | 9 | 34 | 0 | 34 | 55 | M | 178 | 90 |
| **a20** | 511 | 196 | 315 | 9 | 35 | 6 | 41 | 58 | M | 176 | 78 |
| **b01** | 488 | 469 | 19 | 2 | 0.12 | 0.12 | 0.24 | 44 | F | 170 | 63 |
| **b02** | 518 | 425 | 93 | 5 | 14 | 5 | 19 | 53 | M | 176 | 85 |
| **b03** | 442 | 369 | 73 | 4 | 22 | 2 | 24 | 53 | M | 176 | 85 |
| **b04** | 430 | 420 | 10 | 1 | 0.7 | 0 | 0.7 | 42 | M | 180 | 64 |
| **b05** | 434 | 377 | 57 | 3 | 2 | 3 | 5 | 52 | M | 180 | 135 |
| **c01** | 485 | 485 | 0 | 0 | 0 | 0 | 0 | 31 | M | 184 | 74 |
| **c02** | 503 | 502 | 1 | 0 | 0 | 0 | 0 | 37 | M | 180 | 83 |
| **c03** | 455 | 455 | 0 | 0 | 0 | 0 | 0 | 39 | M | 184 | 65 |
| **c04** | 483 | 483 | 0 | 0 | 0 | 0 | 0 | 41 | F | 180 | 65 |
| **c05** | 467 | 464 | 3 | 0 | 0 | 0 | 0 | 28 | F | 169 | 57 |
| **c06** | 469 | 468 | 1 | 0 | 0 | 0.25 | 0.25 | 28 | F | 171 | 65 |
| **c07** | 454 | 450 | 4 | 0 | 0 | 0 | 0 | 30 | F | 168 | 56 |
| **c08** | 535 | 535 | 0 | 0 | 0 | 0 | 0 | 42 | M | 180 | 64 |
| **c09** | 469 | 467 | 2 | 0 | 0 | 0 | 0 | 37 | M | 180 | 83 |
| **c10** | 432 | 431 | 1 | 0 | 0 | 0 | 0 | 27 | M | 184 | 72 |
| **x01** | 524 | 149 | 375 | 9 | 46 | 17 | 63 | 44 | M | 177 | 105 |
| **x02** | 470 | 261 | 209 | 7 | 27.3 | 10.4 | 37.7 | 46 | M | 167 | 69 |
| **x03** | 466 | 454 | 12 | 1 | 0.13 | 0 | 0.13 | 44 | F | 170 | 63 |
| **x04** | 483 | 483 | 0 | 0 | 0 | 0 | 0 | 39 | M | 184 | 65 |
| **x05** | 506 | 190 | 316 | 9 | 34 | 0 | 34 | 55 | M | 178 | 90 |
| **x06** | 451 | 451 | 0 | 0 | 0 | 0 | 0 | 31 | M | 170 | 66 |
| **x07** | 510 | 270 | 240 | 8 | 11 | 10 | 21 | 58 | M | 176 | 78 |
| **x08** | 518 | 194 | 324 | 8 | 48 | 0 | 48 | 55 | M | 178 | 90 |
| **x09** | 509 | 342 | 167 | 5 | 1.8 | 16.7 | 18.5 | 43 | M | 177 | 80 |
| **x10** | 511 | 415 | 96 | 6 | 3 | 7 | 10 | 39 | M | 170 | 131 |
| **x11** | 458 | 445 | 13 | 1 | 2 | 3 | 5 | 52 | M | 180 | 135 |
| **x12** | 528 | 471 | 57 | 4 | 21 | 12 | 33 | 40 | M | 179 | 96 |
| **x13** | 507 | 215 | 292 | 8 | 18.5 | 0.2 | 18.7 | 57 | M | 171 | 97 |
| **x14** | 491 | 52 | 439 | 9 | 61.2 | 18.3 | 79.5 | 38 | M | 180 | 120 |
| **x15** | 499 | 299 | 200 | 8 | 11.3 | 4.6 | 15.9 | 63 | M | 179 | 104 |
| **x16** | 516 | 451 | 65 | 4 | 22 | 2 | 24 | 53 | M | 176 | 85 |
| **x17** | 401 | 400 | 1 | 0 | 0 | 0 | 0 | 27 | F | 158 | 53 |
| **x18** | 460 | 458 | 2 | 0 | 0 | 0 | 0 | 27 | M | 184 | 72 |
| **x19** | 488 | 81 | 407 | 9 | 50.1 | 6.1 | 56.2 | 54 | M | 168 | 80 |
| **x20** | 514 | 250 | 264 | 8 | 34 | 9 | 43 | 51 | M | 179 | 88 |
| **x21** | 511 | 391 | 120 | 4 | 14 | 5 | 19 | 53 | M | 176 | 85 |
| **x22** | 483 | 481 | 2 | 0 | 0 | 0 | 0 | 27 | F | 158 | 53 |

| | | | | | | | | | | | |
|---|---|---|---|---|---|---|---|---|---|---|---|
| **x23** | 528 | 409 | 119 | 3 | 9.9 | 4.4 | 14.3 | 43 | M | 177 | 80 |
| **x24** | 430 | 429 | 1 | 0 | 0 | 0 | 0 | 31 | M | 170 | 66 |
| **x25** | 511 | 220 | 291 | 9 | 48 | 0 | 48 | 55 | M | 178 | 90 |
| **x26** | 521 | 177 | 344 | 9 | 14.4 | 0.7 | 15.1 | 57 | M | 171 | 97 |
| **x27** | 499 | 11 | 488 | 9 | 71 | 4 | 75 | 60 | M | 176 | 113 |
| **x28** | 496 | 62 | 434 | 9 | 71 | 4 | 75 | 60 | M | 176 | 113 |
| **x29** | 471 | 471 | 0 | 0 | 0 | 0 | 0 | 41 | F | 180 | 65 |
| **x30** | 512 | 186 | 326 | 9 | 17 | 24 | 41 | 44 | M | 177 | 105 |
| **x31** | 558 | 42 | 516 | 10 | 86.8 | 6.7 | 93.5 | 29 | F | 183 | 100 |
| **x32** | 539 | 114 | 425 | 9 | 63.3 | 8.5 | 71.8 | 29 | F | 183 | 100 |
| **x33** | 474 | 471 | 3 | 0 | 0.13 | 0 | 0.13 | 28 | F | 169 | 57 |
| **x34** | 476 | 472 | 4 | 0 | 0.38 | 0 | 0.38 | 30 | F | 168 | 56 |
| **x35** | 484 | 484 | 0 | 0 | 0 | 0 | 0 | 31 | M | 184 | 74 |

***S2 Table. Performance metrices for fused images calculated for each fold***

| | ***PR*** | ***RE*** | ***SP*** | ***F1*** |
|---|---|---|---|---|
| 'fold1' | 0.895665 | 0.933384 | 0.929 | 0.914136 |
| 'fold2' | 0.880266 | 0.911945 | 0.919 | 0.895825 |
| 'fold3' | 0.889132 | 0.93267 | 0.924 | 0.910381 |
| 'fold4' | 0.87037 | 0.935681 | 0.909 | 0.901845 |
| 'fold5' | 0.905365 | 0.930322 | 0.9365 | 0.917674 |
| 'fold6' | 0.905344 | 0.908116 | 0.938 | 0.906728 |
| 'fold7' | 0.885901 | 0.933384 | 0.9215 | 0.909023 |
| 'fold8' | 0.882653 | 0.926549 | 0.9195 | 0.904069 |
| 'fold9' | 0.905703 | 0.911945 | 0.938 | 0.908813 |
| 'fold10' | 0.881343 | 0.904288 | 0.9205 | 0.892668 |

***S3 Table. Performance metrices for scalogram images calculated for each fold***

| | ***PR*** | ***RE*** | ***SP*** | ***F1*** |
|---|---|---|---|---|
| 'fold1' | 0.852092 | 0.904288 | 0.8975 | 0.877415 |
| 'fold2' | 0.851695 | 0.92343 | 0.895 | 0.886113 |
| 'fold3' | 0.860769 | 0.908187 | 0.904 | 0.883842 |
| 'fold4' | 0.852541 | 0.911945 | 0.897 | 0.881243 |
| 'fold5' | 0.859021 | 0.900459 | 0.9035 | 0.879252 |
| 'fold6' | 0.86963 | 0.898928 | 0.912 | 0.884036 |
| 'fold7' | 0.847903 | 0.913476 | 0.893 | 0.879469 |
| 'fold8' | 0.866522 | 0.918898 | 0.9075 | 0.891942 |
| 'fold9' | 0.854494 | 0.917305 | 0.898 | 0.884786 |
| 'fold10' | 0.864865 | 0.906585 | 0.9075 | 0.885234 |

***S4 Table. Performance metrices for spectrogram images calculated for each fold***

| | ***PR*** | ***RE*** | ***SP*** | ***F1*** |
|---|---|---|---|---|
| fold1' | 0.870606 | 0.891271 | 0.9135 | 0.880817 |
| 'fold2' | 0.859725 | 0.909717 | 0.903 | 0.884015 |
| 'fold3' | 0.848074 | 0.910413 | 0.8935 | 0.878139 |
| 'fold4' | 0.862518 | 0.912711 | 0.905 | 0.886905 |
| 'fold5' | 0.868479 | 0.905054 | 0.9105 | 0.886389 |
| 'fold6' | 0.846695 | 0.921899 | 0.891 | 0.882698 |
| 'fold7' | 0.850704 | 0.924254 | 0.894 | 0.885955 |
| 'fold8' | 0.850704 | 0.924254 | 0.894 | 0.885955 |
| 'fold9' | 0.848315 | 0.924962 | 0.892 | 0.884982 |
| 'fold10' | 0.836806 | 0.922665 | 0.8825 | 0.87764 |

***S5 Table. Performance metrices for wigner ville distribution images calculated for each fold***

| | **PR** | **RE** | **SP** | **F1** |
|---|---|---|---|---|
| fold1' | 0.653451 | 0.68147 | 0.764 | 0.667166 |
| 'fold2' | 0.807159 | 0.846095 | 0.868 | 0.826168 |
| 'fold3' | 0.797737 | 0.863045 | 0.857 | 0.829107 |
| 'fold4' | 0.732003 | 0.786371 | 0.812 | 0.758213 |
| 'fold5' | 0.805674 | 0.869832 | 0.863 | 0.836524 |
| 'fold6' | 0.785053 | 0.844564 | 0.849 | 0.813722 |
| 'fold7' | 0.784244 | 0.846095 | 0.848 | 0.813996 |
| 'fold8' | 0.815965 | 0.844682 | 0.8755 | 0.830075 |
| 'fold9' | 0.679099 | 0.738897 | 0.772 | 0.707737 |
| 'fold10' | 0.803811 | 0.872129 | 0.861 | 0.836577 |
